# All-optical materials design of chiral edge modes in transition-metal dichalcogenides

**DOI:** 10.1038/ncomms13074

**Published:** 2016-10-10

**Authors:** Martin Claassen, Chunjing Jia, Brian Moritz, Thomas P. Devereaux

**Affiliations:** 1 Department of Applied Physics, Stanford University, Stanford, California 94305, USA; 2 Stanford Institute for Materials and Energy Sciences, SLAC National Accelerator Laboratory and Stanford University, Stanford, California 94025, USA; 3 Department of Physics and Astrophysics, University of North Dakota, Grand Forks, North Dakota 58202, USA; 4 Geballe Laboratory for Advanced Materials, Stanford University, Stanford, California 94305, USA

## Abstract

Monolayer transition-metal dichalcogenides are novel materials which at low energies constitute a condensed-matter realization of massive relativistic fermions in two dimensions. Here, we show that this picture breaks for optical pumping—instead, the added complexity of a realistic materials description leads to a new mechanism to optically induce topologically protected chiral edge modes, facilitating optically switchable conduction channels that are insensitive to disorder. In contrast to graphene and previously discussed toy models, the underlying mechanism relies on the intrinsic three-band nature of transition-metal dichalcogenide monolayers near the band edges. Photo-induced band inversions scale linearly in applied pump field and exhibit transitions from one to two chiral edge modes on sweeping from red to blue detuning. We develop an *ab initio* strategy to understand non-equilibrium Floquet–Bloch bands and topological transitions, and illustrate for WS_2_ that control of chiral edge modes can be dictated solely from symmetry principles and is not qualitatively sensitive to microscopic materials details.

## Abstract (web summary)

Transition metal dichalcogenides offer a platform to study light-matter interaction in atomically thin semiconductors. Here, the authors perform *ab initio* calculations to illustrate the possibility of optical control of chiral edge modes, outlining a strategy to manipulate topological states.

## Graphical abstract

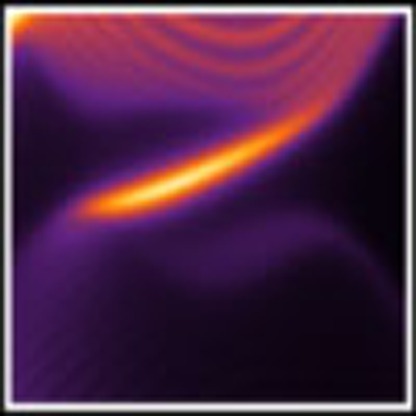

Manipulating materials properties far from equilibrium recently garnered significant attention, with experimental emphasis on transient melting, enhancement, or induction of electronic order^1^,^2^,^3^,^4^. A more tantalizing aspect of the matter–light interaction regards the possibility to access dynamical steady states with distinct non-equilibrium phase transitions to affect electronic transport^5^,^6^,^7^,^8^,^9^. Conceptually simple, irradiation with a sufficiently broad pump pulse dresses the original electronic bands by multiples of the photon frequency, with electric dipole coupling resulting in an effective steady-state band structure; Floquet–Bloch theory then corresponds precisely to the classical limit of strong pump fields that are indistinguishable before and after photon absorption or emission. One paradigmatic model of such Floquet–Bloch bands is graphene^10^,^11^,^12^, where circularly polarized light can break time-reversal symmetry to dynamically lift the Dirac point degeneracies. While Floquet–Bloch states were indeed observed recently via microwave pumping of Dirac cones on the surface of topological insulators^6^,^7^, an extension to proper topological phase transitions is still well beyond experimental reach due to the tremendous required electric field: 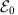 to open a sizeable gap 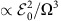 (ref. 10) for above-bandwidth pump frequencies. Conversely, experimentally realizable gaps at the Dirac points at lower pump frequencies^11^,^12^ or in semiconductor quantum wells^5^ come at the price of resonant absorption, heating the sample, or worse, at the required pump strengths.

Viewed naively as semiconducting analogues of graphene, trigonal-prismatic monolayers of MoS_2_, MoSe_2_, WS_2_ and WSe_2_ possess sizeable intrinsic band gaps due to broken inversion symmetry^13^ that can be expected to sustain intense sub-gap pump pulses while limiting absorption for sufficient detuning from the band edge. Prior theoretical studies established that the band edges at **K** and **K**′ are dominated by transition-metal *d*-orbitals, which split into three groups with irreducible representations (IRs) 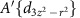, 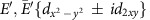 and 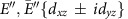 of the *C*_3h_ point group^14^,^15^. Generalizing graphene, these valleys are well-captured in equilibrium by a degenerate Kramers' pair of massive Dirac fermions, giving rise to valley-Hall^13^ and spin-Hall^16^,^17^ effects.

Out of equilibrium, dynamical breaking of time-reversal symmetry was demonstrated to lift the valley degeneracy for WS_2_ and WSe_2_ via off-resonant optical pumping with circularly polarized light^8^,^9^. In this case, the selection rules for a massive Dirac fermion entail that the handedness of pump polarization selectively addresses either the **K** or **K**′ valley, imparting an AC Stark shift on only one of the valleys. Analogously, the photo-excitation can selectively populate valleys, enabling spin and valley currents using circular or linear polarization^18^,^19^,^20^.

Even more tantalizingly, it was predicted that effective TMDC toy models—graphene with a gap—admit, in theory, an optically induced quantum-Hall effect with a single chiral mode localized at the sample edge. A high-frequency pump Ω→∞ well above the bandwidth can in principle close and invert the equilibrium band gap at a single valley^21^; however, this requires a tremendous pump intensity. Alternatively, it was proposed that a resonant pump beam can hybridize the massive Dirac fermion valence and conduction bands (CBs), and thereby generate a single chiral edge mode^8^ at lower pump strength.

In this study, we instead show that such a simple description fails to hold for optical pumping; here, the added complexity of a more realistic model of TMDC monolayers opens up a novel avenue to engineer a Floquet topological insulator in a realistic experimental setting. We argue that correctly addressing optical excitations necessitates a minimally three-band description near the band edges that leads to a frequeny-tunable mechanism to photo-induce one or two chiral edge modes. To understand the nature of concurrent Floquet band inversions at both **K** and **K**′, we develop an *ab initio* Floquet **k**.**p** formalism that directly connects equilibrium density-functional theory (DFT) calculations with non-equilibrium Floquet theory. We illustrate these predictions for the example of a WS_2_ ribbon, and present non-equilibrium ribbon spectra as well as an *ab initio* characterization of the photo-induced valley topological band inversions. We find that control of chiral edge modes is determined solely by crystal symmetry and is insensitive to materials microscopics such as multi-photon processes or local inter-orbital dipole transitions that cannot be captured straightforwardly in a tight-binding model.

## Results

### Three-band nature of the light–matter interaction

**Figure 1:**
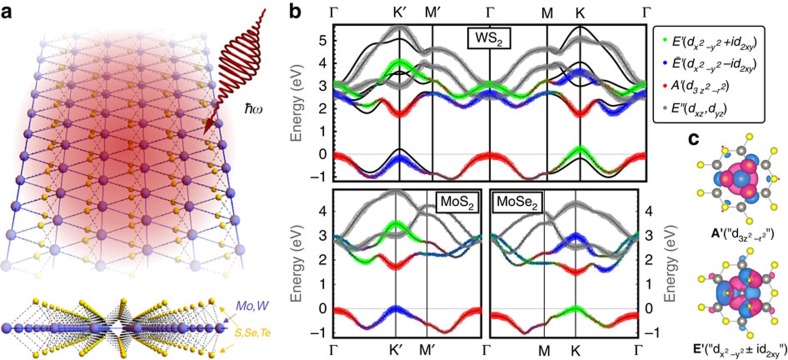
*Ab initio* electronic structure of transition-metal dichalcogenide monolayers. (**a**) Setup: a circularly polarized pump beam irradiates a monolayer transition-metal dichalcogenide ribbon. (**b**) Band structure and orbital content for MoS_2_ and MoSe_2_, as well as WS_2_ with spin–orbit coupling, determined from *ab initio* DFT calculations and downfolding onto localized Wannier orbitals. K and K' are related by time-reversal symmetry. (**c**) Isosurfaces highlight that the effective Wannier orbitals for WS_2_, while localized on W, take into account orbital content extending to the S atoms as well as neighbouring W atoms.

Central to this study, dipole transitions to higher-lying bands, as determined directly from *ab initio* calculations, and underlying symmetry considerations are crucial for a determination of photo-induced topological band inversions in TMDCs. To understand the breakdown of a description as graphene with a gap, consider a monolayer ribbon uniformly irradiated by circularly polarized light, with collinear sample and polarization planes (Fig. 1a). In graphene, the low-energy bands near the Fermi level are separated from higher-energy CBs by >10 eV at **K**,**K**′ (ref. 22), hence optical frequencies can be treated safely within the canonical low-energy Dirac model of **π** orbitals. In contrast, the band structures of prototypical TMDC monolayers possess a *E*′ band only ∼2 eV above the CB (Fig. 1b), and E′′ bands in the same vicinity^23^,^24^,^25^,^26^. Circularly polarized light at close-to-band gap pump frequencies therefore couples the *A*′ CB to both the *E*′ valence band (VB) and the 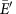 higher-energy CB (XB), while leaving the *E*′′ bands decoupled in the absence of multipole transitions.

**Figure 2:**
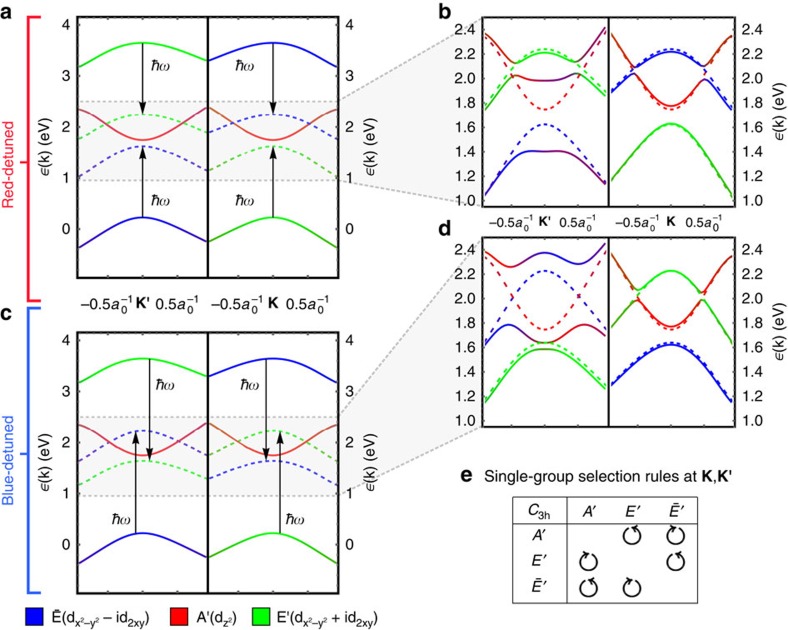
Photo-induced hybridization gaps and band inversion at K,K'. Photon-dressed *ab initio* Floquet bands of Kramer's pair **K**,**K**', for red (**a**,**b**) and blue (**c**,**d**) detuning. A red-detuned (blue-detuned) pump generically brings into resonance a ring of states of the *E*′ higher-energy band (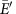 VB) with the *A*′ CB, while leaving the VB (higher-energy band) off-resonant. Electric dipole coupling mixes the equilibrium orbital characters at **K**,**K**' while lifting degeneracies between the CB and photon-dressed copies of the other bands (**b**,**d**). The ensuing photo-induced hybridization gap leads to topologically non-trivial band inversion at a single valley. (**e**) The corresponding *C*_3h_ single group selection rules at **K**,**K**' for circular polarization (see Supplementary Note 1, table 2 for a double group generalization).

Consider first the case of slightly red-detuned pumping below the band edge (Fig. 2a). Here, a ring of states from the higher-energy XB is brought into resonance with the CB, while simultaneously avoiding resonant coupling between the VB and CB. Central to experimental feasability, this regime can be expected to substantially limit absorption and heating. At the band edge, *C*_3h_ dipole selection rules (Fig. 2e) dictate that absorption of a photon couples transitions 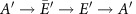. At **K**′, the bare CB |*m*=0; *A*′, C*B*〉 (Floquet index *m*) couples to the XB dressed by a single emitted photon 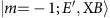 as well as the VB dressed by a single absorbed photon 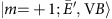. Both transitions, although off-resonant, are energetically favourable, leading to a significant Stark shift at **K**′ (Fig. 2b). Conversely, at **K** the IRs of VB and XB are reversed. Here, the CB couples to the VB dressed by a single emitted photon |*m*=−1; *E*′, V*B*〉 as well as the XB dressed by a single absorbed photon 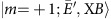. Both transitions are energetically unfavourable, leading to a negligible shift of the band edge. Slightly away from **K** and **K**′, electric dipole coupling lifts the ring of degeneracy between the CB and XB and opens a photo-induced hybridization gap at both valleys (Fig. 2b), which scales linearly with weak pump fields. Crucially, the resulting Floquet–Bloch bands exhibit a topological ‘band inversion' with the orbital character flipped close to the valley minimum, at both valleys (Fig. 2b).

### Topology of photo-induced band inversions

To discern whether the band inversions can be non-trivial, we devise effective Floquet two-band models of the hybridization gaps. We start from the generic description 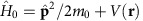 of a semiconductor in the absence of spin–orbit coupling and excitonic effects, where *V*(**r**) is the crystal potential. In equilibrium, starting from an *ab initio* Bloch eigenbasis at a single high-symmetry point, the dispersion and orbital content follow from canonical **k**.**p** theory by perturbing in momentum deviation **k** under replacement 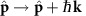 (ref. 27). In the presence of a time-periodic field **A**(*t*)=*A*[cos(Ω*t*), sin(*ωt*)]^T^, a straight-forward generalization uses a Floquet eigenbasis of the generic non-equilibrium problem 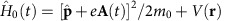. This basis can be obtained from DFT calculations via knowledge of the equilibrium band energies and dipole transition matrix elements. Note that this Floquet **k**.**p** theory is non-perturbative in the applied pump field and naturally accounts for multi-photon coupling to higher-energy CBs, XBs and deeper VBs, as well as local inter-orbital dipole transitions. In the following, 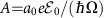 denotes the dimensionless field strength with lattice constant *a*_0_=3.2 Å and electric field 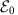; *A*=0.1 corresponds to 
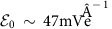
 for optical pump fields with Ω=1.5 eV. An effective low-energy description of the photo-induced gaps can now be devised in Floquet basis in analogy to the equilibrium problem, by considering a perturbation in crystal momentum 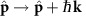 and downfolding onto effective two-band Floquet models using canonical Löwdin perturbation theory.

Central to the robustness of this proposal, the form of these effective models is determined solely from symmetry and is universal to trigonal-prismatic TMDC monolayers. To see this, first consider **K**. Here, the Floquet eigenbasis (Fig. 2b) |Ψ_1_〉 (|Ψ_2_〉) admixes |*m*=0; *A*′, C*B*〉 with |*m*=−1; *E*′, V*B*〉, 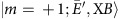 (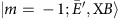 with |*m*=−2; *A*′, C*B*〉, |*m*=0; *E*′, V*B*〉), linear in field *A*. Constrained by crystal symmetry, we find that the effective Floquet physics at **K** is generically determined by a *p*-*d* Dirac model (see the ‘Methods' section):


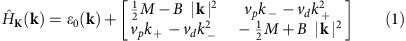


An additional purely dispersive term 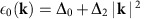 breaks particle-hole symmetry. Crucially, the off-diagonal couplings *v*_*p*_,*v*_*d*_ are linear functions in field strength, suggesting a sizable photo-induced gap already at weak fields. While the parameters depend on the details of the Bloch states near the Dirac points, overall topological considerations can be gleaned simply from equation (1). In the absence of *v*_*d*_, equation (1) describes a conventional massive Dirac fermion, with *M* (*B*) the Dirac (inverse band) mass, and *v*_*p*_ the Dirac velocity. The orbital character exhibits a *p*-wave winding around **K** and mirrors the quantum anomalous Hall effect in Hg_*y*_Mn_1–_*_y_*Te quantum wells^28^. Switching on *v*_*d*_ imparts a trigonal distortion by reducing the continuous rotational symmetry around **K** to *C*_3_, and introduces instead a ‘*d*-wave' winding in the limit 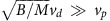.

At **K**′ interchanged IRs 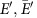 entail a strongly admixed Floquet eigenbasis as well as a significant Stark shift. However selection rules forbid a coupling between |Ψ_1_〉 ,|Ψ_2_〉 linear in **k**—instead, one finds that the two bands couple to quadratic order in 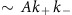, via intermediate states |*m*=0; *E*′, *XB*〉 or |*m*=−1; *A*′, *CB*〉. The effective Hamiltonian for **K**′ in this case generically reads


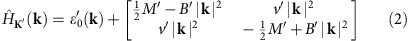


with *v*′ a rotationally symmetric band mixing term.

At **K** (**K**′), the band ordering is inverted when *M*/*B*>0 (*M*′/*B*′>0). If the orbital character of Floquet–Bloch bands in the remaining Brillouin zone is sufficiently benign, we can draw conclusions on the global topology by understanding separately the band inversions at **K** and **K**′. Rewriting equations (1) and (2) in terms of Pauli matrices 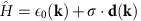, the local Berry curvature follows from the winding 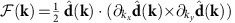 with 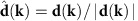. One can see by inspection that the absence of 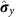 in equation (2) enforces 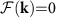 ; therefore, the photo-induced band inversion around **K**′ is necessarily trivial. Conversely, the band inversion at **K** is topological and triggers a change in the Chern number 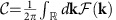, which captures the contribution to topology arising from the band inversion in the vicinity of **K** (see Supplementary Note 3, Supplementary Fig. 1).

Consider first the limit of a massive Dirac model with *v*_*d*_=0. In this case, the Chern number changes from 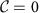 for *B*/*M* < 0 to 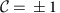 for *B*/*M*>0, inducing a single chiral edge mode, spanning the photo-induced hybridization gap, and localized at the boundary of a uniformly illuminated sample. Switching on *v*_*d*_ introduces a trigonal distortion of the Floquet–Bloch bands around **K** up to a critical strength 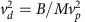, at which the Floquet–Bloch bands close the gap at three points away from **K**, related by *C*_3_. Correspondingly, this topological transition changes 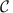 by 3, to 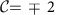, entailing not one but two chiral edge modes at the sample boundary.

### Red versus blue detuning

To get an understanding of the mechanism that might trigger this transition, note that while the relevant Floquet basis is predominantly built from only the *m*=0 CB and *m*=−1 XB for a red-detuned pump, the *p*-wave coupling *v*_*p*_ between the two is necessarily mediated via the VB (or other bands of equivalent IR), highlighting the necessity of a minimal three-band description (see Supplementary Note 2). We stress that such an effective Dirac-like contribution is generically impossible to obtain starting from a two-band equilibrium description as a massive Dirac fermion. In contrast, the *d*-wave term results from direct coupling between CB and XB. Strong optical absorption in TMDC monolayers indicates a large dipole transition matrix element between VB and CB, suggesting that a sufficiently red-detuned pump will generically reach the *C*=1 phase. Note that the asymmetry between **K**,**K**′ stems from the choice of polarization, and reverses for opposite handedness of the circularly polarized pump beam.

In contrast, consider the opposite regime of a sufficiently blue-detuned pump (Fig. 2c). Here, a ring of VB states is brought into resonance with the CB near **K**,**K**′ while pushing the photon-dressed XB into the equilibrium band gap. Electric dipole coupling again opens photo-induced hybridization gaps, both at the bottom of the CB and top of the VB (Fig. 2d), and the symmetry analysis mirrors the discussion of the red-detuned case above, leading to equivalent effective Hamiltonians at **K**,**K**′ (1), (2). However, linear in **k** coupling between the *m*=0 CB and the *m*=+1 VB is now necessarily mediated via the XB (or other 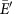 bands separated further in energy), whereas the *d*-wave term *v*_*d*_ follows directly from dipole coupling between VB and CB, dominating over *v*_*p*_. One can thus generically expect a frequency-tunable Floquet Chern insulator in monolayer TMDCs.

### *Ab initio* Floquet analysis of WS_2_ monolayers

**Figure 3:**
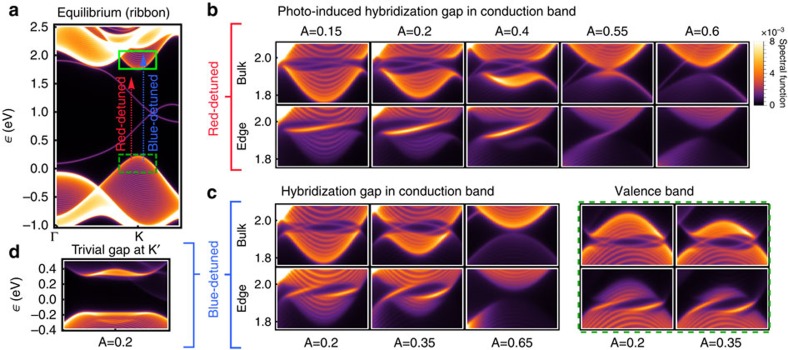
Single-particle spectra for a WS_2_ ribbon irradiated with circularly polarized light. (**a**) In equilibrium, the ribbon hosts two trivial edge states localized at opposing edges. (**b**) A red-detuned pump field induces a hybridization gap at the bottom of the CB, which scales linearly in *A* until closing again at a critical pump strength. (**c**) A blue-detuned pump couples the equilibrium valence and CBs, inducing gaps both at the bottom of the conduction and top of the VB, each hosting two chiral edge modes. (**d**) Hybridization gaps at the opposite valley **K**' lead merely to a trivial band inversion, depicted here for the top of the VB for blue detuning.

To illustrate these predictions, consider WS_2_ as a prototypical TMDC monolayer. First, we perform *ab initio* DFT calculations to derive an effective minimal tight-binding model of three 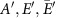 Wannier orbitals localized on the W transition metal (Fig. 1b,c). The resulting Floquet spectrum on a ribbon is depicted in Fig. 3, calculated from the period-averaged single-particle spectral function (see the ‘Methods' section). In equilibrium (Fig. 3a), WS_2_ already hosts a pair of trivial edge states in analogy to zigzag edges of graphene, with right (left) propagating modes at the **K** (**K**′) point that span the band gap. A weak, red-detuned pump field opens a hybridization gap at the bottom of the CB, spanned by a single chiral mode at **K** (Fig. 3b), localized at the sample edge. The photo-induced gap scales linearly with weak *A*, but closes and reopens at a critical pump strength, transitioning again to a trivial phase without chiral modes. Conversely, for a blue-detuned pump (Fig. 3c) a second chiral edge mode appears, spanning the hybridization gaps both at the bottom of the CB and the top of the VB.

**Figure 4:**
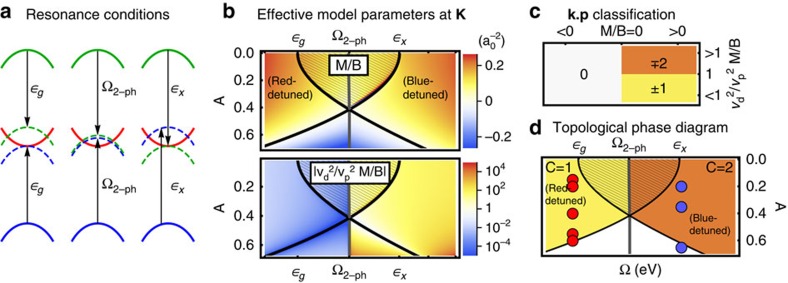
Topological band inversions and Floquet k.p theory in a Wannier three-orbital model of WS_2_. (**a**) The relevant resonances when moving from red to blue detuning. (**b**) Effective **k**.**p** parameterization of the band inversion at **K** (Eqn. 1) starting from the three-orbital Wannier tight-binding model. (**c**) sketches the corresponding topological classification. At low pump strengths, the sign of *M*/*B a*_0_^ 2^ is negative in the non-trivial inverted regime. The system transitions from one (red-detuned) to two (blue-detuned) chiral edge modes when 
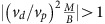
. The effective **k**.**p** model parameterized in **b** accounts for the Floquet state adiabatically derived from the CB, as well as the Floquet state deriving from the VB dressed by one absorbed photon, for red detuning, or from the higher-energy *E*′ XB dressed by one emitted photon, for blue detuning. This entails the sharp transition when sweeping the pump frequency across the two-photon resonance between VB and XB (**a**). Within the shaded areas, all three bands come into resonance. Thick black lines indicate degeneracies in the Floquet spectrum. (**d**) A corresponding numerical calculation of the global Chern number of the tight-binding model mirrors the **k**.**p** analysis. Yellow (orange) denotes 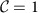 (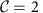) as defined in **c**, circles indicate parameters used for Fig. 2.

The appearances of edge modes in ribbon spectra are in excellent agreement with effective model parameters (equations (1) and (2)) derived from the Wannier tight-binding description. Figure 4b depicts *M*/*B* and the ratio of *p*−/*d*−wave couplings that determine the Chern number for the band inversion at **K** (Fig. 4c), in perfect correspondence with a rigorous calculation of the 2+1D Floquet winding number^29^ of the driven Wannier tight-binding model (Fig. 4d, see Supplementary Note 3), with the circularly polarized pump entering via Peierls substitution. For weak fields, deep within both the red- and blue-detuned regimes, the sign of the Dirac *M* and band *B* mass are equal in the topologically non-trivial phase. Here, *C*≠0, and the Chern number follows from trigonal distortion and changes from *C*=1 for red detuning to *C*=2 for blue detuning. Increasing *A* instead closes and reopens the Floquet gap at **K**, flips the sign of *M* and uninverts the bands to reach a trivial phase with *C*=0. We note that this picture breaks down for intense pump fields with energy scales on the order of the equilibrium band gap; here, additional topological phase transitions can arise (Supplementary Fig. 2, Note 4).

**Figure 5:**
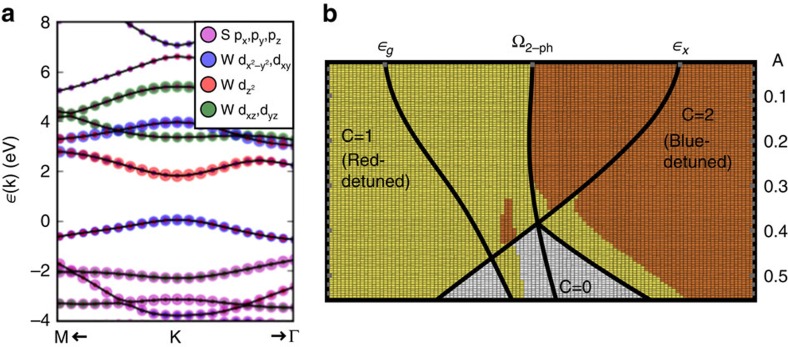
*Ab initio* Floquet k.p classification of photo-induced band inversions in WS_2_. (**a**) Starting from a 185-band first-principles description of the **K** point, multi-photon processes involving deep core levels and higher-energy as well as local dipole transitions can be taken into account exactly. (**b**) Recomputing the effective Floquet **k**.**p** two-band model of the photo-induced band inversion recovers a phase diagram that is qualitatively similar to the three-band tight-binding description (Fig. 4d), suggesting that the photo-induction of one or two chiral edge modes for appropriate tuning of the pump laser is robust to multi-photon processes at weak pump fields. Strong pump-field deviations indicate a non-trivial admixing of higher-energy bands.

Having checked the validity of Floquet **k**.**p** theory in the tight-binding model, we now turn to the full *ab initio* problem. To quantify the effects of multi-photon resonances, as well as local inter-orbital dipole transitions not captured in a tight-binding model, we consider an *ab initio* 185-band description of band energies and dipole transition matrix elements at **K** and **K**′ and calculate the model parameters of equations (1) and (2), taking into account up to four-photon processes. The bands closest to the equilibrium gap are depicted in Fig. 5a. The resulting **k**.**p** classification is depicted in Fig. 5b. Crucially, while the resonance lines distort due to effects not accounted for in the tight-binding model, the frequency-dependent switch from *C*=1 to *C*=2 as well as the reclosing of the hybridization gap and transition back to a trivial regime with increasing pump strength remain qualitatively similar. This suggests that the mechanism of photo-induced chiral edge modes described in this work is largely robust at weak fields to the microscopic details of the material.

## Discussion

A key challenge for a condensed-matter realization of Floquet topological insulators regards driving the system strongly to induce the required changes to the equilibrium band structure while simulteanously mitigating the inevitable absorption and heating in such a scheme. However, a common thread for pioneering works on graphene^11^,^12^, semiconductor quantum wells^5^ and topological insulator surface states^6^,^7^ is that these require either high-frequency pumping at tremendous pump strengths or resonant pumping, thereby injecting substantial energy into the system and heating the sample. Recent work has tried to tackle these problems by discerning whether special couplings to bosonic or fermionic heat sinks^30^,^31^,^32^ can dissipate enough energy and nevertheless stabilize a Floquet steady-state ensemble. Analogously, the blue-detuned regime entails a ring of resonance between CB and VB, leading to enhanced absorption. Conversely, and central to the experimental feasability of this proposal, red-detuned pumping of monolayer TMDCs circumvents these issues by entirely avoiding resonant coupling between VB and CB while nevertheless inducing non-trivial band topology by virtue of the minimally three-band nature of electron–photon coupling. Naively, this can be understood by noting that, for weak recombination rates, the rate of carrier photo-excitation scales as *g*^2^*A*^2^/*δ*^2^ whereas the photo-induced hybridization gap scales linearly in pump strength *A* (see the ‘Methods' section), where *δ* is the laser detuning from resonance and *g* an effective dipole matrix element. Nevertheless, this residual photo-excited population will lead to broadening of the Floquet–Bloch bands due to recombination and electron–phonon scattering and set a lower bound on observable bulk hybridization gaps, while at the same time serving as a necessary ingredient to reach a steady-state population. Extending the topological characterization to open quantum systems in the limit of strong dissipation remains an interesting topic for future study^33^.

Smoking-gun evidence for the presence of chiral edge modes necessitates either measurement of the sample edge via nano angle-resolved photoemission (nano-ARPES) and local admittance probes such as microwave impedance spectroscopy, or direct transport measurements, scaling of conductance with sample length, where care must be taken regarding coupling between leads and the chiral Floquet edge mode^34^,^35^. Conversely, ARPES stands as an immediate tool to verify the predicted photo-induced hybridization gaps in the 2D bulk, as a function of pump strength and when sweeping from red to blue detuning. For red detuning, the CB can be enhanced in two-photon photoemission, in analogy to probing the unoccupied higher-energy topological surface states of Bi_2_Se_3_ (ref. 36). An interesting follow-up question concerns potential matrix element dependencies of photoemission from the W *d*-orbitals, to directly observe and characterize the band inversions at **K**,**K**′ via bulk measurement.

The guiding theme of this work has been to build a bridge between the rapidly developing field of monolayer transition-metal dichalcogenides and topological phase transitions out of equilibrium, to provide a route towards achieving the latter in an experimentally attainable setting. We have shown that the three-band nature of the valleys in prototypical WS_2_ leads to a new mechanism to ‘switch' on or off, one or two chiral edges with near band gap optical irradiation. The resulting photo-induced gap in the single-particle spectrum scales linearly with pump strength, suggesting substantial energy scales already at low fields, while simultaneously ensuring minimal heating with sufficient detuning from the band edge. Our theoretical analysis of the out-of-equilibrium valley band inversions connects directly with equilibrium *ab initio* calculations, whereas the ensuing topology of Floquet–Bloch bands relies purely on generic symmetry arguments, suggesting that the predictions are robust to microscopic detail and should be observable in a range of monolayer TMDC materials. Finally, our first-principles and theoretical analysis provides a promising strategy to predict and design topological states out of equilibrium in other semiconductor materials.

## Methods

### *Ab initio* calculations

*Ab initio* calculations were performed in the framework of the Perdew–Burke–Ernzerhof type generalized gradient approximation of DFT using the full-potential linearized augmented plane wave method implemented in Wien2k (ref. 37). We consider a single monolayer of WS_2_ with a 30Å vacuum space perpendicular to the layer along the *z*-direction. The in-plane lattice constant and the S position have been relaxed by optimization of the total energy and total force, respectively. For electronic structure calculations, we utilized a 15 × 15 × 1 *k*-space grid. Momentum matrix element calculations were performed using the OPTIC package implemented in Wien2k, with a 60 × 60 × 1 *k*-space grid. Maximally localized Wannier functions for the five W 5*d* orbitals were obtained using wien2wannier (ref. 38) and Wannier90 (ref. 39) with initial projections set to the spherical harmonics *Y*_2*m*_ (*m*=−2, −1, 0, 1, 2). Due to the symmetry of the hexagonal lattice, the calculated Hamiltonian in the new Wannier basis naturally decouples into the two standard subspaces {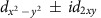, 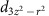} and {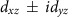}.

### Floquet theory of the single-particle spectrum on a ribbon

Floquet theory captures the effective steady states that arise from a time-dependent (quasi-)periodic modulation. Consider a Hamiltonian 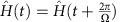 with a periodic time dependence with frequency Ω. Then, solutions of the time-dependent Schrödinger equation for 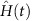 can be written as 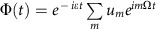, where 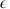 is the Floquet quasi-energy, and *u*_*m*_ are Fourier coefficients of the time-periodic part of the wave function. Substitution of Φ(*t*) into Schrödinger's equation recasts the time-dependent problem as an effective time-independent Floquet problem: the Floquet states can be found by finding eigenstates of the Floquet Hamiltonian


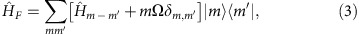


where, 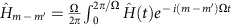 are the Fourier expansion coefficients of 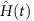. If the original Hamiltonian has a static eigenbasis |*α*〉, then the eigenstates of 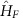 can be written as 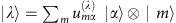, with the original time-dependent eigenstates of 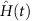 becoming 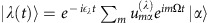. The next step is to connect back to observables of the original fermion operators. In the main text, we consider the spectral function


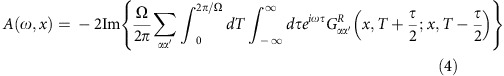


where, 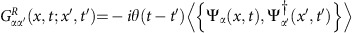 is the retarded Green's function. Rewriting the fermion operators Ψ_*α*_(*x*,*t*) in Floquet basis, one finally arrives at the Floquet spectral function


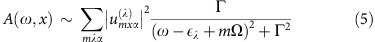


where Γ is a phenomenological broadening of the spectrum.

### Floquet **k.p** theory and effective Hamiltonians at **K**,**K′**

Consider a generic time-dependent starting point for **K** (and equivalently for **K**′)


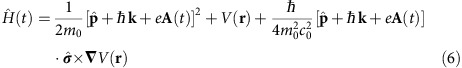


where, **k** is the deviation in crystal momentum from **K** or **K**′, with a respective shift to the **K** or **K**′ point absorbed in 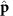. We consider circularly polarized light with **A**(*t*)=*A*[cos(Ω*t*), sin(Ω*t*)]^T^. Now decompose 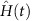 into equilibrium [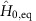] and non-equilibrium [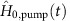] constituents that determine the eigenbasis at **K**,**K**′, as well as a perturbation in **k** [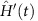]:


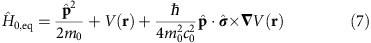



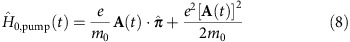



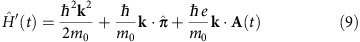


where, 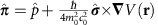. In equilibrium, the (time-independent) eigenbasis |*α*,*n*〉 of 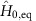 can be determined from *ab initio* calculations and transforms according to *C*_3h_, with *n*,*α* indexing the nth band with IR *α*. In the absence of the pump field **A**(*t*)=0, conventional **k**.**p** theory proceeds by considering the deviation in Bloch momentum as a perturbation, described by 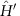.

In Floquet **k**.**p** theory, one instead starts from the exact Floquet eigenbasis of 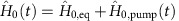 at **K** and **K**′. Consider a single spin manifold, and for simplicity denote bands by the *C*_3h_ single group IRs 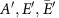 (see Supplementary Table 2, Supplementary Note 1 for equivalent double group identifications and selection rules in the full SOC problem). The selection rules (Fig. 2e, Supplementary Table 1) then entail that 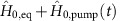 involve transitions


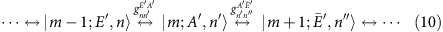


Here, *m* (*n*) are Floquet (band) indices, and 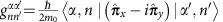 are the momentum matrix elements for allowed dipole transitions (Fig. 2e), obtained from *ab initio* calculations. Using a sufficiently large number of *ab initio*-determined Bloch states at **K**,**K**′, their dipole matrix elements and Floquet side bands, the Floquet eigenbasis at **K**,**K**′ can formally be determined exactly as functions of *A*,Ω.

The effective two-band Hamiltonians (equations (1) and (2)) described in the main text now follow via choosing two Floquet eigenstates for **K** and **K**′ each, that are adiabatically connected to the *A*=0 CB with *m*=0 as well as the *m*=−1XB (*m*=+1VB) for red (blue) detuning, and using Löwdin perturbation theory to downfold 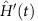 onto this two-state Floquet eigenbasis (a detailed derivation can be found in Supplementary Note 2).

Crucially, to distinguish **K** and **K**′, note that their irreducible representations 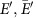 for VB, XB interchange. A simple way to arrive at the effective Hamiltonians (1) and (2) follows from observing for (10) that the Floquet eigenbasis at **K**,**K**′ necessarily decomposes again into three IRs of the joint electron–photon problem, which are subsequently coupled by the Bloch momentum perturbation 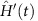. In this picture, at **K**, the IRs of the two Floquet basis states of (1) differ; hence off-diagonal coupling enters already at linear order 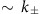. Note that this coupling can necessarily only arise in the present minimally three-band description (see Supplementary Material). Conversely, at **K**′ the IRs of the basis of (2) are the same; off-diagonal coupling therefore necessarily enters only to quadratic order 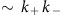 and higher, leading to a trivial band inversion.

The inclusion of full spin–orbit coupling does not qualitatively alter these conclusions. First, spin-flip terms weakly admix *E*′′ bands of opposite spin^40^,^41^; however, the full crystal double group 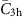 again decomposes into two spin-orbital manifold with equivalent selection rules and effective physics (Supplementary Note 1). Second, the valley Zeeman shift simply leads to a shift of the relevant resonance energies. Similarly, while monolayer TMDCs have been shown to give rise to large excitonic binding energies^42^,^43^, in the context of our work their role is confined to shifting the relevant resonance energies, given appropriate tuning of the pump frequency.

### Data availability

The data that support the findings of this study are available from the authors on request.

## Additional information

**How to cite this article**: Claassen, M. *et al*. All-optical materials design of chiral edge modes in transition-metal dichalcogenides. *Nat. Commun.* **7**, 13074 doi: 10.1038/ncomms13074 (2016).

## Supplementary Material

Supplementary Information Supplementary Figures 1-2, Supplementary Tables 1-2, Supplementary Notes 1-4 and Supplementary References
