# Supplementary material for: All-optical materials design of chiral edge modes in transition-metal dichalcogenides
Source: Nat Commun. 2016 Oct 10;7:13074. doi: 10.1038/ncomms13074 (PMC5062430; doi:10.1038/ncomms13074)
Supplement: Supplementary Information — Supplementary Figures 1-2, Supplementary Tables 1-2, Supplementary Notes 1-4 and Supplementary References [file ncomms13074-s1.pdf]

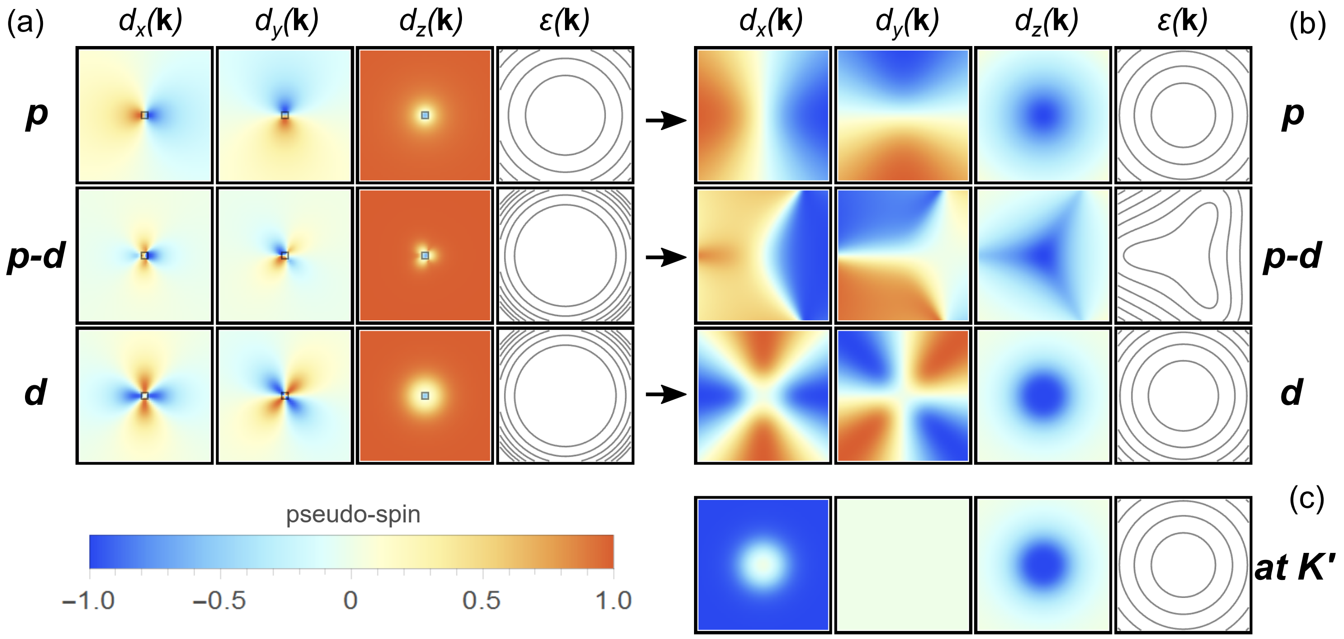

Supplementary Figure 1: **Floquet-Bloch pseudospin textures at K and K′.** (a) At **K** the pseudospin texture interpolates between p-wave ($v_p \neq 0, v_d = 0$) and d-wave ($v_p = 0, v_d \neq 0$) winding upon increase of trigonal distortion. Given *a priori* knowledge that the band structure remains gapped globally, a topological invariant can be assigned by introducing a regularizer η that ensures that the pseudospin points into the z direction for $k \to \infty$ and the **k**-space manifold can thus be compactified to a sphere. Trigonal distortion is seen clearly when zooming into the vicinity (black squares) of the **K** point, shown enlarged in (b). Here, while the local p-d wave texture appears distorted, globally either p-wave or d-wave winding is recovered as seen in (a). At $\mathbf{K}'$, the pseudospin is trivial, with a vanishing y-component, depicted in (c).

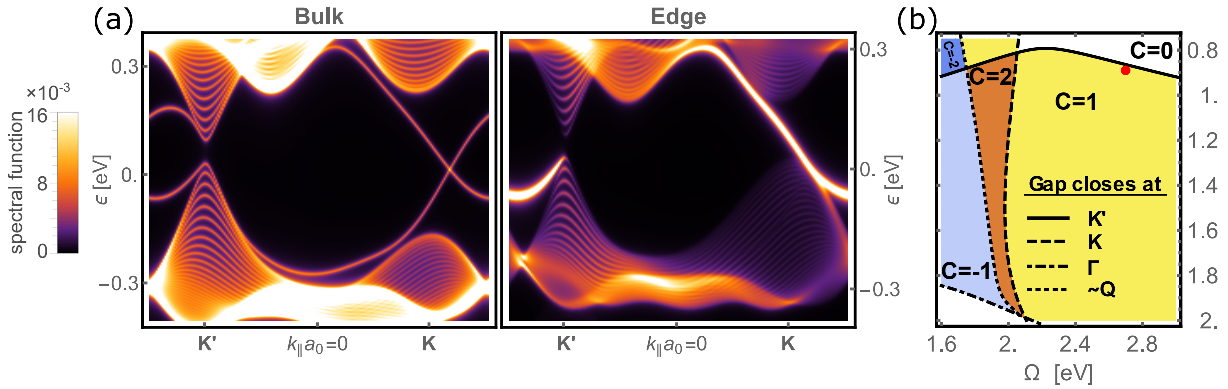

Supplementary Figure 2: **Photo-induced inversions in WS_2 for strong pump fields.** (a) A sufficiently blue-detuned pump field closes the equilibrium gap selectively at a single valley $\mathbf{K}'$, transitioning into a $\mathcal{C} = 1$ phase. One of the two trivial equilibrium edge states disappears, leaving a single chiral edge mode that spans the band gap. The corresponding phase diagram of Floquet Chern numbers at strong pump strengths is depicted in (b), with (a) corresponding to parameters $A = 0.9, \omega = 2.7eV$. Further increase of the pump amplitude or decrease of frequency triggers additional gap closings at **K**, **Γ**, and around the second conduction band minimum **Q**, inducing a mosaic of possible Chern numbers for the photo-modulated valence band. Closing the gap at **K**, $\mathbf{K}'$ changes the Chern number by ± 1, whereas C_3 symmetry dictates closing the gap at **Q** must happen at three points in the Brillouin zone, triggering a change of the Chern number by ± 3. Progressive flattening of the valence band dispersion at strong pump fields indicates the onset of Wannier-Stark physics.

Supplementary Table 1: The single group $C_{3\mathrm{h}}$

(a) $C_{3\mathrm{h}}$ single group character table, with $\Omega = \exp(2\pi i/3)$.

	E	C_3^+	C_3^-	σ_h	S_3^+	S_3^-	invariants
A'	1	1	1	1	1	1	$x^2 + y^2,\ z^2$
E'	1	Ω	$\bar{\Omega}$	1	Ω	$\bar{\Omega}$	$x - iy,\ (x + iy)^2$
$\bar{E}'$	1	$\bar{\Omega}$	Ω	1	$\bar{\Omega}$	Ω	$x + iy,\ (x - iy)^2$
A''	1	1	1	-1	-1	-1	z
E''	1	Ω	$\bar{\Omega}$	-1	-Ω	-$\bar{\Omega}$	$(x - iy)z$
$\bar{E}''$	1	$\bar{\Omega}$	Ω	-1	-$\bar{\Omega}$	-Ω	$(x + iy)z$

(b) Selection rules for electric dipole transitions, for circular polarization

	A'	E'	$\bar{E}'$
A'		↺	↻
E'	↻		↺
$\bar{E}'$	↺	↻	

Supplementary Table 2: The double group $\bar{C}_{3\mathrm{h}}$

(a) $\bar{C}_{3\mathrm{h}}$ double group character table, with complex characters $\Omega = \exp(2\pi i/3)$.

	E	C_3^+	C_3^-	σ_h	S_3^+	S_3^-	$\bar{E}$	$\bar{C}_3^+$	$\bar{C}_3^-$	$\bar{\sigma}_h$	$\bar{S}_3^+$	$\bar{S}_3^-$	invariants
Γ_1	1	1	1	1	1	1	1	1	1	1	1	1	x^2+y^2, z^2
Γ_2	1	Ω	$\bar{\Omega}$	1	Ω	$\bar{\Omega}$	1	Ω	$\bar{\Omega}$	1	Ω	$\bar{\Omega}$	$x-iy, (x+iy)^2$
Γ_3	1	$\bar{\Omega}$	Ω	1	$\bar{\Omega}$	Ω	1	$\bar{\Omega}$	Ω	1	$\bar{\Omega}$	Ω	$x+iy, (x-iy)^2$
Γ_4	1	1	1	-1	-1	-1	1	1	1	-1	-1	-1	z
Γ_5	1	Ω	$\bar{\Omega}$	-1	$-\Omega$	$-\bar{\Omega}$	1	Ω	$\bar{\Omega}$	-1	$-\Omega$	$-\bar{\Omega}$	$(x-iy)z$
Γ_6	1	$\bar{\Omega}$	Ω	-1	$-\bar{\Omega}$	$-\Omega$	1	$\bar{\Omega}$	Ω	-1	$-\bar{\Omega}$	$-\Omega$	$(x+iy)z$
Γ_7	1	$-\Omega$	$-\bar{\Omega}$	i	$-i\Omega$	$i\bar{\Omega}$	-1	Ω	$\bar{\Omega}$	$-i$	$i\Omega$	$-i\bar{\Omega}$	$\uparrow_z$
Γ_8	1	$-\bar{\Omega}$	$-\Omega$	$-i$	$i\bar{\Omega}$	$-i\Omega$	-1	$\bar{\Omega}$	Ω	i	$-i\bar{\Omega}$	$i\Omega$	$\downarrow_z$
Γ_9	1	$-\Omega$	$-\bar{\Omega}$	$-i$	$i\Omega$	$-i\bar{\Omega}$	-1	Ω	$\bar{\Omega}$	i	$-i\Omega$	$i\bar{\Omega}$	
Γ_{10}	1	$-\bar{\Omega}$	$-\Omega$	i	$-i\bar{\Omega}$	$i\Omega$	-1	$\bar{\Omega}$	Ω	$-i$	$i\bar{\Omega}$	$-i\Omega$	
Γ_{11}	1	-1	-1	i	$-i$	i	-1	1	1	$-i$	i	$-i$	
Γ_{12}	1	-1	-1	$-i$	i	$-i$	-1	1	1	i	$-i$	i	

(b) single-group and double-group irreducible representations of the Wannier orbital basis

state	single group IR	double group IR
$\lvert d_{3z^2-r^2}, \uparrow\rangle$	A'	Γ_7
$\lvert d_{x^2-y^2} - id_{2xy}, \uparrow\rangle$	E'	Γ_{10}
$\lvert d_{x^2-y^2} + id_{2ixy}, \uparrow\rangle$	$\bar{E}'$	Γ_{11}
$\lvert d_{xz-iyz}, \uparrow\rangle$	E''	Γ_8
$\lvert d_{xz+iyz}, \uparrow\rangle$	$\bar{E}''$	Γ_{12}
$\lvert d_{3z^2-r^2}, \downarrow\rangle$	A'	Γ_8
$\lvert d_{x^2-y^2} - id_{2xy}, \downarrow\rangle$	E'	Γ_{12}
$\lvert d_{x^2-y^2} + id_{2xy}, \downarrow\rangle$	$\bar{E}'$	Γ_9
$\lvert d_{xz-iyz}, \downarrow\rangle$	E''	Γ_{11}
$\lvert d_{xz+iyz}, \downarrow\rangle$	$\bar{E}''$	Γ_7

(c) Selection rules for electric dipole transitions, for circular polarization

$\bar{C}_{3\mathrm{h}}$ IR		$\Uparrow$			$\Downarrow$		
		Γ_7	Γ_{10}	Γ_{11}	Γ_8	Γ_{12}	Γ_9
	Γ_7		$\circlearrowleft$	$\circlearrowright$			
$\Uparrow$	Γ_{10}	$\circlearrowright$		$\circlearrowleft$			
	Γ_{11}	$\circlearrowleft$	$\circlearrowright$				
	Γ_8					$\circlearrowleft$	$\circlearrowright$
$\Downarrow$	Γ_{12}				$\circlearrowright$		$\circlearrowleft$
	Γ_9				$\circlearrowleft$	$\circlearrowright$	

Supplementary Note 1 Symmetry Analysis and the Role of Spin-Orbit Coupling

In the absence of spin-orbit coupling (SOC), the relevant bands near $\mathbf{K}, \mathbf{K}'$ can be classified according to the single-group irreducible representations (IRs) of the point group $C_{3\mathrm{h}}$, denoted $A', E', \bar{E}', E'', \bar{E}''$ [1, 2]. The corresponding character table and relevant invariants are depicted in Supplementary Table 2. As discussed in the main text, reflection symmetry σ_h guarantees that bands $A', E', \bar{E}'$ remain decoupled from $E'', \bar{E}'$ across the entire Brillouin zone [3, 4, 5, 6, 7], and the analysis can therefore be constrained to three relevant bands $A', E', \bar{E}'$ only. In 2H monolayer TMDCs, the conduction band transforms as A' and is dominantly composed of the transition metal $d_{3z^2-r^2}$ orbital, whereas the valence and relevant higher-energy band transform as $E', \bar{E}'$ with a dominant contribution of $d_{x^2-y^2} \pm id_{2xy}$ orbitals. Furthermore, spin z is a good quantum number.

In equilibrium, the band structure can be expanded in $\mathbf{k}$ around $\mathbf{K}, \mathbf{K}'$ by starting from Hamiltonian

$$\hat{H} = \hat{H}_0 + \hat{H}_{\mathrm{SOC}} + \hat{H}_{\mathbf{k}} \tag{S1}$$

where

$$\hat{H}_0 = \frac{1}{2m_0}\hat{\mathbf{p}}^2 + V(\mathbf{r}) \tag{S2}$$

$$\hat{H}_{\mathrm{SOC}} = \frac{\hbar}{4m_0^2c_0^2}\hat{\mathbf{p}} \cdot \hat{\boldsymbol{\sigma}} \times \boldsymbol{\nabla} V(\mathbf{r}) \tag{S3}$$

$$\hat{H}_{\mathbf{k}} = \frac{\hbar^2\mathbf{k}^2}{2m_0} + \frac{\hbar}{2m_0}\mathbf{k} \cdot \left[\hat{\mathbf{p}} + \frac{\hbar}{4m_0^2c_0^2}\hat{\boldsymbol{\sigma}} \times \boldsymbol{\nabla} V(\mathbf{r})\right] \tag{S4}$$

Here, $\hat{\boldsymbol{\sigma}}$ are the Pauli matrices, and $V(\mathbf{r})$ is the crystal potential.

The role of SOC can now be understood in two complementary ways, by either considering the eigenstates of $\hat{H}_0$ as IRs of the $C_{3\mathrm{h}}$ single group and treating SOC as a perturbation, or by starting from the true Bloch eigenstates of $\hat{H}_0 + \hat{H}_{\mathrm{SOC}}$ at $\mathbf{K}, \mathbf{K}'$, as IRs of the $\bar{C}_{3\mathrm{h}}$ double group. In the former case, the eigenstates of $\hat{H}_0$ are spin-z eigenstates with a given single-group IR, namely $|A'[d_{3z^2-r^2}], \sigma\rangle$, $\left|E'[d_{x^2-y^2} + id_{2xy}], \sigma\right\rangle$ and $\left|\bar{E}'[d_{x^2-y^2} - id_{2xy}], \sigma\right\rangle$ with $\sigma =\uparrow_z, \downarrow_z$. To understand the effect of SOC, it is useful to decompose $\hat{H}_{\mathrm{SOC}}, \hat{H}'_{\mathbf{k}}$:

$$\hat{H}^z_{\mathrm{SOC}} = \hat{\sigma}_z \left[\hat{p}_x\partial_y - \hat{p}_y\partial_x\right] V(\mathbf{r}) \tag{S5}$$

$$\hat{H}^{\uparrow\downarrow}_{\mathrm{SOC}} = 2i\hat{\sigma}^- \left[\hat{p}_z\partial_+V(\mathbf{r}) - \hat{p}_+\partial_zV(\mathbf{r})\right] + \text{h.c.} \tag{S6}$$

$$\hat{H}^z_{\mathbf{k}} = \frac{\hbar^2\mathbf{k}^2}{2m_0} + \frac{\hbar}{2m_0}\left[k_+ \cdot \left(\hat{\mathbf{p}} - i\hat{\sigma}_z\frac{\hbar}{4m_0^2c_0^2}\partial_-V(\mathbf{r})\right) + \text{h.c.}\right] \tag{S7}$$

$$\hat{H}^{\uparrow\downarrow}_{\mathbf{k}} = \frac{\hbar}{2m_0}k_+ \cdot \left(\hat{\mathbf{p}} + i\hat{\sigma}_-\frac{\hbar}{4m_0^2c_0^2}\partial_zV(\mathbf{r})\right) + \text{h.c.} \tag{S8}$$

Here, $\hat{H}^z_{\mathrm{SOC}}$ transforms as A' and acts as a mere Zeeman shift, whereas the spin-flip contribution $\hat{H}^{\uparrow\downarrow}_{\mathrm{SOC}}$ transforms as $E'', \bar{E}''$ and hence couples states with opposite parity under σ_h. The latter entails a mixing between conduction band state $|A', \sigma\rangle$ and $\left|E''(\bar{E}''), -\sigma\right\rangle$ as well as between the higher-energy conduction band $\left|\bar{E}', \uparrow\right\rangle$ and $|E'', \downarrow\rangle$ while leaving the opposite spin $\left|\bar{E}', \downarrow\right\rangle$ unmixed. This mixing of $\uparrow_z, \downarrow_z$ states is however suppressed since the energy difference between the A', E' and E'' bands is larger than the spin-orbit coupling, and the SOC at $\mathbf{K}, \mathbf{K}'$ can be well-captured as an effective Zeeman shift while approximately leaving spin as a good quantum number [5, 7, 8, 9, 10]. Away from $\mathbf{K}, \mathbf{K}'$, $\hat{H}^z_{\mathbf{k}}$ transforms as $E', \bar{E}'$ and imparts an additional momentum-dependent Zeeman shift. Conversely, $\hat{H}^{\uparrow\downarrow}_{\mathbf{k}}$ transforms as A'', which does not couple the A' states of the original conduction band while weakly mixing the E', E'' bands.

Consider now the double group, with its character table given in Supplementary Table 2. Spin-flip mixing with the $E'', \bar{E}''$ bands necessarily reduces the number of band IRs from 5 IRs $\times 2$ spin orientations,

to 6 double-group IRs. These again decompose into two manifolds denoted $\Uparrow, \Downarrow$ that remain decoupled over the entire Brillouin zone. The identification of band states with single group and double group IRs is given in Supplementary Table 2(b). Note that the states listed are not an eigenbasis of $\hat{H}_0 + \hat{H}_{\mathrm{SOC}}$: instead, the true eigenbasis will be a superposition of states of equal double-group IR, governed by the strength of SOC. At $\mathbf{K}, \mathbf{K}'$, one can immediately deduce that the eigenbasis entails mixing of $|d_{3z^2-r^2}, \sigma\rangle$ and $|d_{xz+\sigma\cdot iyz}, -\sigma\rangle$, of $\left|d_{x^2-y^2+2ixy} + id_{2xy}, \uparrow\right\rangle$ and $|d_{xz-iyz}, \downarrow\rangle$, and of$|d_{xz+iyz}, \uparrow\rangle$ and $\left|d_{x^2-y^2} - id_{2ixy}, \downarrow\right\rangle$, while leaving states $\left|d_{x^2-y^2} - id_{2xy}, \uparrow\right\rangle$ and $\left|d_{x^2-y^2} + id_{2xy}, \downarrow\right\rangle$ unmixed and as proper eigenstates of $\hat{H}_0 + \hat{H}_{\mathrm{SOC}}$.

Here, the $\mathbf{k.p}$ perturbation transforms as Γ_2, Γ_3 and couples the three $\Uparrow$ IRs $\Gamma_7, \Gamma_{10}, \Gamma_{11}$ ($\Downarrow$ IRs $\Gamma_8, \Gamma_9, \Gamma_{12}$) in an equivalent manner as in the single-group case of $A', E', \bar{E}'$ without spin-orbit coupling, or with SOC but without spin-flip terms. Consequently, the selection rules of electric dipole transitions for circular polarization are exactly equivalent, as shown in Supplementary Table 2(c). For this reason, we chose to simply adopt the single-group notation and label the spin manifolds as $\uparrow_z, \downarrow_z$, while keeping in mind that SOC is indeed significant for certain TMDCs and enters through a one-to-one correspondence with the double group IRs and spin manifolds $\Uparrow, \Downarrow$.

Supplementary Note 2 Floquet $k.p$ Theory

A central result of the main text, the effective Floquet $\mathbf{k.p}$ Hamiltonians (1) and (2), as determined from symmetry considerations of the non-equilibrium problem, characterize the photo-induced band inversions at $\mathbf{K}$ and $\mathbf{K}'$ and describe transitions between zero, one and two chiral edge modes as a function of pump strength and frequency. To further shed light on the procedure of Floquet $\mathbf{k.p}$ theory as outlined in the Methods section, we consider here an explicit derivation of Eqns. (1) and (2) of the main text, in the limit of small A.

We set $\hbar = 1$. The first step concerns finding the time-dependent Floquet eigenbasis directly at $\mathbf{K}, \mathbf{K}'$, as eigenfunctions of $\hat{H}_{\mathrm{eq}} + \hat{H}_{\mathrm{pump}}(t)$ [Eq. (7), (8) in the Methods section]. Working with $C_{3\mathrm{h}}$ IRs, this equivalently amounts to solving the time-independent Floquet eigenbasis of the Floquet Hamiltonian:

$$\begin{aligned}
\hat{H}_{0F} = &\sum_{mn\alpha} \left(\epsilon_{n\alpha} + m\Omega + \frac{e^2A^2}{2m_0}\right) |m;\alpha n\rangle\langle m;\alpha n| \\
&+ eA \sum_{mnn'} \Big(g_{nn'}^{A'E'} \left|m+1; A', n\right\rangle\left\langle m; E', n'\right| \\
&\qquad + g_{nn'}^{\bar{E}'A'} \left|m+1; \bar{E}', n\right\rangle\left\langle m; A', n'\right| \\
&\qquad + g_{nn'}^{E'\bar{E}'} \left|m+1; E', n\right\rangle\left\langle m; \bar{E}', n'\right| + \text{h.c.}\Big)
\end{aligned} \tag{S9}$$

Here, m is the Floquet index; n, α index the nth band in the $C_{3\mathrm{h}}$ IRs $\alpha = A', E', \bar{E}'$, and $g_{nn'}^{\alpha\alpha'} = \frac{1}{2m_0}\langle \alpha, n|(\hat{\boldsymbol{\pi}}_x - i\hat{\boldsymbol{\pi}}_y)|\alpha', n'\rangle$ are the momentum matrix elements for allowed dipole transitions that are obtained from *ab initio* calculations.

Having determined the new eigenbasis of (S9) that admixes different Floquet side bands and IRs of $C_{3\mathrm{h}}$, deviations in momentum away from $\mathbf{K}, \mathbf{K}'$ can now be treated as a perturbation in $\hat{H}'(t)$ [Eq. (9) in the Methods section]:

$$\begin{aligned}
\hat{H}' = k_+ \Bigg\{ &\sum_{mn\alpha} \frac{e}{m_0}\frac{A}{2} |m-1;\alpha,n\rangle\langle m;\alpha,n| \\
&+ \sum_{mnn'} \Big(g_{nn'}^{A'E'} \left|m; A', n\right\rangle\left\langle m; E', n'\right| \\
&\qquad + g_{nn'}^{\bar{E}'A'} \left|m; \bar{E}', n\right\rangle\left\langle m; A', n'\right| \\
&\qquad + g_{nn'}^{E'\bar{E}'} \left|m; E', n\right\rangle\left\langle m; \bar{E}', n'\right| \Big) \Bigg\} + \text{h.c.}
\end{aligned} \tag{S10}$$

At $\mathbf{K}$, and in the limit of small A, the Floquet eigenstates of $\hat{H}_{0F}$ that compose the eigenbasis of Eq. (1) of the main text can be found perturbatively:

$$|\Psi_{\mathbf{K},1}\rangle = \big|0; A', \mathrm{CB}\big\rangle + eA\left[\sum_n \frac{(g^{A'E'}_{\mathrm{CB},n})^\star\ \big|{-1}; E', n\big\rangle}{\epsilon_{A',\mathrm{CB}} - \epsilon_{E',n} + \Omega} + \sum_n \frac{g^{\bar{E}'A'}_{n,\mathrm{CB}}\ \big|{+1}; \bar{E}', n\big\rangle}{\epsilon_{A',\mathrm{CB}} - \epsilon_{\bar{E}',n} - \Omega}\right] + \mathcal{O}(A^2) \tag{S11}$$

$$|\Psi_{\mathbf{K},2}\rangle = \big|{-1}; \bar{E}', \mathrm{XB}\big\rangle + eA\left[\sum_n \frac{(g^{\bar{E}'A'}_{\mathrm{XB},n})^\star\ \big|{-2}; A', n\big\rangle}{\epsilon_{\bar{E}',\mathrm{XB}} - \epsilon_{A',n} + \Omega} + \sum_n \frac{g^{E'\bar{E}'}_{n,\mathrm{XB}}\ \big|0; E', n\big\rangle}{\epsilon_{\bar{E}',\mathrm{XB}} - \epsilon_{E',n} - \Omega}\right] + \mathcal{O}(A^2) \tag{S12}$$

In a second step, one can now start from this eigenbasis and perturb in $\mathbf{k}$, which amounts to a perturbation in $\hat{H}'$ [Eqns. (9), (11) in the Methods section]. Here, a coupling between $|\Psi_1\rangle$, $|\Psi_2\rangle$ is mediated via the $m = 0; E'$ VB (and via all other bands of E' IR) to linear order $\sim Ak_-$, as well as via the $m = -1; A'$ CB or the $m = 0; \bar{E}'$ XB to quadratic order $\sim Ak_+^2$. To second order in $\mathbf{k}$, we arrive at the effective Hamiltonian (1) quoted in the main text, where, to linear order in A, we obtain p- and d-wave couplings of the form

$$v_p = eA \sum_n g^{A'E'}_{\mathrm{CB},n}\ g^{E'\bar{E}'}_{n,\mathrm{XB}} \left(\frac{1}{\epsilon_{A',\mathrm{CB}} - \epsilon_{E',n} + \Omega} + \frac{1}{\epsilon_{\bar{E}',\mathrm{XB}} - \epsilon_{E',n} - \Omega}\right) + \mathcal{O}(A^2) \tag{S13}$$

$$\begin{aligned} v_d = eA \Bigg\{ &\frac{1}{4m_0}\ (g^{\bar{E}'A'}_{\mathrm{XB},\mathrm{CB}})^\star \left(\frac{1}{\epsilon_{A',\mathrm{CB}} - \epsilon_{\bar{E}',\mathrm{XB}}} - \frac{1}{\Omega} + \frac{1}{\Omega} + \frac{1}{\epsilon_{\bar{E}',\mathrm{XB}} - \epsilon_{A',\mathrm{CB}}}\right) + \\ &+ \frac{1}{2}\sum_{nn'} \frac{g^{E'\bar{E}'}_{n,\mathrm{XB}} \left(g^{E'\bar{E}'}_{nn'}\, g^{\bar{E}'A'}_{n',\mathrm{CB}}\right)^\star}{\epsilon_{\bar{E}',\mathrm{XB}} - \epsilon_{E',n} - \Omega} \left(\frac{1}{\epsilon_{A',\mathrm{CB}} - \epsilon_{\bar{E}',n'}} + \frac{1}{\epsilon_{E',n} - \epsilon_{\bar{E}',n'}}\right) + \\ &+ \frac{1}{2}\sum_{nn'} \frac{g^{A'E'}_{\mathrm{CB},n'} \left(g^{\bar{E}'A'}_{\mathrm{XB},n} g^{A'E'}_{nn'}\right)^\star}{\epsilon_{A',\mathrm{CB}} - \epsilon_{E',n'} + \Omega} \left(\frac{1}{\epsilon_{E',n'} - \epsilon_{A',n}} + \frac{1}{\epsilon_{\bar{E}',\mathrm{XB}} - \epsilon_{A',n}}\right)\Bigg\} + \mathcal{O}(A^2) \end{aligned} \tag{S14}$$

together with Dirac and band mass terms

$$M = \epsilon_{\bar{E}',\mathrm{XB}} - \epsilon_{A',\mathrm{CB}} - \Omega + \underbrace{\mathcal{O}(A^2)}_{\text{Stark shift}} \tag{S15}$$

$$B = \sum_{n\gamma}\left[\frac{\left|g^{\gamma,A'}_{n,\mathrm{CB}}\right|^2}{\epsilon_{A',\mathrm{CB}} - \epsilon_{n,\gamma}} - \frac{\left|g^{\gamma,\bar{E}'}_{n,\mathrm{XB}}\right|^2}{\epsilon_{\bar{E}',\mathrm{XB}} - \epsilon_{n,\gamma}}\right] + \mathcal{O}(A^2) \tag{S16}$$

As can be seen from the above derivations, an essential consequence of the minimally three-band description of electron-photon coupling in TMDCs is the appearance of the linear "Dirac" coupling v_p on the level of the effective Floquet Hamiltonian for $\mathbf{K}$. Indeed, v_p necessarily depends on dipole matrix elements that couple all three bands $\sim g^{A'E'}_{\mathrm{CB},n}\ g^{E'\bar{E}'}_{n,\mathrm{XB}}$ with n the VB or further bands of E' IR. Therefore, besides naturally precluding the appearance of a red-detuned regime, equilibrium two-band toy models of TMDCs can never capture a non-equilibrium transition between $C = 2$ and $C = 1$ in the blue-detuned regime.

Conversely, at $\mathbf{K'}$, the perturbative Floquet eigenstates of $\hat{H}_{0F}$ that compose the eigenbasis of Eq. (2) in the main text read:

$$\big|\Psi_{\mathbf{K'},1}\big\rangle = \big|0; A', \mathrm{CB}\big\rangle + eA\left[\sum_n \frac{(g^{A'E'}_{\mathrm{CB},n})^\star\ \big|{-1}; E', n\big\rangle}{\epsilon_{A',\mathrm{CB}} - \epsilon_{E',n} + \Omega} + \sum_n \frac{g^{\bar{E}'A'}_{n,\mathrm{CB}}\ \big|{+1}; \bar{E}', n\big\rangle}{\epsilon_{A',\mathrm{CB}} - \epsilon_{\bar{E}',n} - \Omega}\right] + \mathcal{O}(A^2) \tag{S17}$$

$$\big|\Psi_{\mathbf{K'},2}\big\rangle = \big|{-1}; E', \mathrm{XB}\big\rangle + eA\left[\sum_n \frac{(g^{E'\bar{E}'}_{\mathrm{XB},n})^\star\ \big|{-2}; \bar{E}', n\big\rangle}{\epsilon_{E',\mathrm{XB}} - \epsilon_{\bar{E}',n} + \Omega} + \sum_n \frac{g^{A'E'}_{n,\mathrm{XB}}\ \big|0; A', n\big\rangle}{\epsilon_{E',\mathrm{XB}} - \epsilon_{A',n} - \Omega}\right] + \mathcal{O}(A^2) \tag{S18}$$

Here, $\left|\Psi_{\mathbf{K}',1}\right\rangle$ and $\left|\Psi_{\mathbf{K}',2}\right\rangle$ remain decoupled to linear order in $\mathbf{k}$. Instead, we arrive at a quadratic coupling of the form $\sim Ak_+k_- = A\mathbf{k}^2$ as discussed in the main text, with

$$
\begin{aligned}
v' = eAg^{A',E'}_{\mathrm{CB,XB}} \frac{1}{4m_0} &\left(\frac{1}{\epsilon_{A',\mathrm{CB}} - \epsilon_{E',\mathrm{XB}}} - \frac{1}{\Omega} + \frac{1}{\Omega} + \frac{1}{\epsilon_{E',\mathrm{XB}} - \epsilon_{A',\mathrm{CB}}} \right) + \\
&+ \sum_{nn'} eA \frac{g^{A'E'}_{n',\mathrm{XB}}}{\epsilon_{E',\mathrm{XB}} - \epsilon_{A',n'} - \Omega} \left[g^{\bar{E}'A'}_{n,n'} (g^{\bar{E}'A'}_{n,\mathrm{CB}})^\star \left(\frac{1}{\epsilon_{A',\mathrm{CB}} - \epsilon_{\bar{E}',n}} + \frac{1}{\epsilon_{A',n} - \epsilon_{\bar{E}',n'}} \right) + \right. \\
&\left. + (g^{A'E'}_{n'n})^\star g^{A'E'}_{\mathrm{CB},n} \left(\frac{1}{\epsilon_{A'\mathrm{CB}} - \epsilon_{E',n}} + \frac{1}{\epsilon_{A',n'} - \epsilon_{E',n}} \right) \right] + \\
&+ \sum_{nn'} eA \frac{g^{A'E'}_{\mathrm{CB},n}}{\epsilon_{A',\mathrm{CB}} - \epsilon_{E',n} + \Omega} \left[g^{A'E'}_{n',\mathrm{XB}} (g^{A'E'}_{n',n})^\star \left(\frac{1}{\epsilon_{E',n} - \epsilon_{A',n'}} + \frac{1}{\epsilon_{E',\mathrm{XB}} - \epsilon_{A',n'}} \right) + \right. \\
&\left. + (g^{E'\bar{E}'}_{\mathrm{XB},n'})^\star g^{E'\bar{E}'}_{n,n'} \left(\frac{1}{\epsilon_{E',n} - \epsilon_{\bar{E}',n'}} + \frac{1}{\epsilon_{E',\mathrm{XB}} - \epsilon_{\bar{E}',n'}} \right) \right]
\end{aligned}
\tag{S19}
$$

Supplementary Note 3 Topological Classification and Pseudospin Textures

The main text classifies photo-induced topological phase transitions via local effective Floquet $\mathbf{k}.\mathbf{p}$ Hamiltonians at $\mathbf{K}, \mathbf{K}'$. The key idea is to understand the global topology via a local classification of band inversions at $\mathbf{K}, \mathbf{K}'$, which relies on *a-priori* knowledge that 1) the Floquet spectrum is gapped globally and 2) the Berry curvature behaves benign at other high-symmetry points in the Brillouin zone. Armed with this knowledge, a complementary view of local band inversions follows from considering so-called pseudospin textures around $\mathbf{K}$ and $\mathbf{K}'$. Starting from the local Floquet $\mathbf{k}.\mathbf{p}$ Hamiltonians $\hat{H}_{\mathbf{K}}(\mathbf{k})$ and $\hat{H}_{\mathbf{K}'}(\mathbf{k})$ of equations 1 and 2 of the main text, we can recast these in terms of pseudospin Pauli matrices $\boldsymbol{\sigma}$:

$$
\hat{H}_{\nu=\mathbf{K},\mathbf{K}'}(\mathbf{k}) = \epsilon_0(\mathbf{k}) + \epsilon(\mathbf{k})\ \hat{\mathbf{d}}_\nu(\mathbf{k}) \cdot \boldsymbol{\sigma} \tag{S20}
$$

Here, $\epsilon_0(\mathbf{k}) \pm \epsilon(\mathbf{k})$ is the dispersion of $\hat{H}_{\mathbf{K}}(\mathbf{k})$ or $\hat{H}_{\mathbf{K}'}(\mathbf{k})$, and $\hat{\mathbf{d}}_\nu(\mathbf{k})$ is the pseudospin vector, with $|\hat{\mathbf{d}}_\nu(\mathbf{k})| = 0$. Here, $\nu = \mathbf{K}, \mathbf{K}'$. The pseudospin vector equivalently follows from taking the expectation value $\langle \boldsymbol{\sigma} \rangle$ of the Floquet-Bloch states around $\mathbf{K}, \mathbf{K}'$.

As discussed in the main text, the band inversion at $\mathbf{K}'$ is trivial, and the pseudospin obeys $d^y_{\mathbf{K}}(\mathbf{k}) = 0$. Conversely, at $\mathbf{K}$, the pseudospin becomes

$$
\hat{\mathbf{d}}_{\mathbf{K}}(\mathbf{k}) = \frac{1}{N(\mathbf{k})} \left[v_p k_x + v_d(k_x^2 - k_y^2),\ v_p k_y - 2 v_d k_x k_y,\ M - B|\mathbf{k}|^2 \right]^\top \tag{S21}
$$

with a normalization $N(\mathbf{k})$ to ensure that $|\hat{\mathbf{d}}_{\mathbf{K}}(\mathbf{k})| = 1$.

At $\mathbf{K}$, the effective Hamiltonian S20 can be viewed as a d-wave generalization of the conventional massive Dirac Hamiltonian, with an additional band mass term in analogy to the $\mathbf{k}.\mathbf{p}$ model at Γ for HgTe/CdTe quantum wells. Supplementary Figure 1 depicts the pseudospin textures upon increasing v_d/v_p to enhance trigonal distortion. $v_p \neq 0, v_d = 0$ recovers the conventional massive Dirac fermion with a quadratic band mass term; here, the pseudospin has a p-wave winding around $\mathbf{K}'$ and the Chern number becomes $C = 1$. In the opposite limit $v_p = 0, v_d \neq 0$, the pseudospin acquires a d-wave winding around $\mathbf{K}$. Here, since the band mass term is quadratic only, the winding in $d^x(\mathbf{k}), d^y(\mathbf{k})$ persists in principle to $k \to \infty$. Given the knowledge that the band structure is gapped globally and that the band inversion should be confined to high-symmetry points, this behavior is an artifact of the lower-order $\mathbf{k}.\mathbf{p}$ expansion.

More rigorously, quantization of the integral $\mathcal{C} = \frac{1}{2\pi} \int_{\mathbb{R}^2} d\mathbf{k} \mathcal{F}(\mathbf{k})$ necessitates a compactification of k-space $\mathbb{R}^2$ to a non-contractible manifold. This can be motivated as follows: Since the Floquet $\mathbf{k}.\mathbf{p}$ theory can be expected to faithfully represent the physics only in the close vicinity around $\mathbf{K}$, momenta

$\mathbf{k}$ far away from $\mathbf{K}$ should not affect $\mathcal{C}$. This can be enforced rigorously by adding to $\hat{H}$ an infinitesimal rotationally-symmetric regularizer $-\eta B'\hat{\sigma}_z|\mathbf{k}|^4$ with $\eta \to 0$, leading to:

$$\hat{\mathbf{d}}^{(\text{reg})}_{\mathbf{K}'}(\mathbf{k}) = \frac{1}{N(\mathbf{k})}\left[v_p k_x + v_d(k_x^2 - k_y^2),\ v_p k_y - 2v_d k_x k_y,\ M - B|\mathbf{k}|^2(1+\eta|\mathbf{k}|^2)\right]^\top \tag{S22}$$

It follows that the unit vector $\hat{\mathbf{d}}^{(\text{reg})}_{\mathbf{K}'}(|\mathbf{k}| \to \infty) = -\text{sgn}(B)\hat{e}_z$ does not depend on the polar angle θ of $\mathbf{k}$, and $\mathbb{R}^2$ can be compactified to a sphere S_2 by identifying ∞ with the north pole without loss of information. The choice $\hat{\sigma}_z$ of the regularizer is motivated by noting that $|B| \gg |v_d|$ for any choice of pump field entails that $\hat{\mathbf{d}}(|\mathbf{k}| \to \infty) \sim -\text{sgn}(B)\hat{e}_z + \frac{v_d}{|B|}[\cos(2\theta)\hat{e}_x - \sin(2\theta)\hat{e}_y]$ already approximately points in the z-direction.

The band inversion can be seen clearly by looking at the behavior of $d^z(\mathbf{k})$ close to $\mathbf{K}$, which switches sign when going from $\mathbf{k} = 0$ to $\mathbf{k} \to \infty$. The intermediate regime of p-d-wave winding leads to a distorted pseudospin texture when looking at the close vicinity of $\mathbf{K}$, whereas a d-wave (or p-wave) texture is retained at large $\mathbf{k}$ to arrive at a $C = 2$ or $C = 1$ phase.

Supplementary Note 4 Strong Pumping and Inversion of the Equilibrium Band Gap

Discussions on photo-induced chiral edge states have focused so far solely on dynamically-generated gaps within the equilibrium conduction and valence bands, since a sizeable energy scale $\sim 1.5eV$ in WS_2 protects the equilibrium band gap from closing for weak pump fields. This picture changes conceivably when approaching the regime of Wannier-Stark physics at significantly higher pump strength. In the high-frequency limit, broken time-reversal symmetry then bestows an optical Stark shift of equal and opposite magnitude on $\mathbf{K}$ and $\mathbf{K}'$, that bridges the equilibrium band gap at a critical field strength A. The gap closes and reopens at $\mathbf{K}'$ to eliminate one branch of the trivial equilibrium edge states, leaving a single chiral edge state to bridge the Floquet-Bloch band gap at $\mathbf{K}$, as depicted in Supplementary Figure 2(a). Upon even further increase of A, the gap finally closes and reopens at Γ, returning to a trivial regime without chiral edge modes. Naïvely, the flattening of the bands upon crossover to Wannier-Stark ladders at increasing pump strengths A or decreasing frequencies Ω suggests that one should not expect to continue attributing special significance to the original high-symmetry points in this regime. Nevertheless, the system can undergo a series of gap closings confined to $\mathbf{K}, \mathbf{K}', \Gamma$ upon further decrease of Ω. The associated topological transitions change the Floquet Chern number by ± 1, leading to a mosaic of photo-induced topological phases at high pump intensities. In addition, gap closures occur near the second conduction-band minimum $\mathbf{Q}$ in WS_2. As this is not a high-symmetry point, C_3 rotation symmetry dictates that the gap must instead close simultaneously at three distinct points in the Brillouin zone, changing the Chern number by ± 3. We verified the corresponding phase diagram in Supplementary Figure 2(b) by numerically evaluating the Floquet Chern number.

Supplementary References

[1] Bromley, R.A., Murray, R.B. & Yoffe, A.D. The band structures of some transition metal dichalcogenides. III. group via: Trigonal prism materials. *J. of Physics C: Solid State Phys.* 5, 759 (1972).

[2] Mattheiss, L.F. Band structures of transition-metal-dichalcogenide layer compounds. *Phys. Rev. B* 8, 3719 (1973).

[3] Xiao, D., Liu, G.-B., Feng, W., Xu, X. & Yao, W. Coupled spin and valley physics in monolayers of MoS_2 and other group-VI dichalcogenides. *Phys. Rev. Lett.* 108, 196802 (2012).

[4] Zahid, F., Liu, L., Zhu, Y., Wang, J. & Guo, H. A generic tight-binding model for monolayer, bilayer and bulk MoS_2. *AIP Advances* 3, 052111 (2013).

[5] Cappelluti, E., Roldán, R., Silva-Guillén, J.A., Ordejón, P. & Guinea, F. Tight-binding model and direct-gap/indirect-gap transition in single-layer and multilayer MoS_2. *Phys. Rev. B* 88, 075409 (2013).

[6] Rostami, H., Moghaddam, A.G. & Asgari, R. Effective lattice hamiltonian for monolayer MoS_2: Tailoring electronic structure with perpendicular electric and magnetic fields. *Phys. Rev. B* 88, 085440 (2013).

[7] Liu, G.-B., Shan, W.-Y., Yao, Y., Yao, W. & Xiao, D. Three-band tight-binding model for monolayers of group-VIB transition metal dichalcogenides. *Phys. Rev. B* 88, 085433 (2013).

[8] Gibertini, M., Pellegrino, F.M.D., Marzari, N. & Polini, M. Spin-resolved optical conductivity of two-dimensional group-VIB transition-metal dichalcogenides. *Phys. Rev. B* 90, 245411 (2014).

[9] Kormányos, A. *et al.* Monolayer MoS_2: Trigonal warping, the γ valley, and spin-orbit coupling effects. *Phys. Rev. B* 88, 045416 (2013).

[10] Feng, W. *et al.* Intrinsic spin hall effect in monolayers of group-VI dichalcogenides: A first-principles study. *Phys. Rev. B* 86, 165108 (2012).
